# Primary medulla oblongata germinomas: two case reports and review of the literature

**DOI:** 10.1186/1477-7819-11-274

**Published:** 2013-10-15

**Authors:** Shuyu Hao, Da Li, Jie Feng, Liang Wang, Zhen Wu, Junting Zhang, Liwei Zhang

**Affiliations:** 1 Department Neurosurgery, Beijing Tiantan Hospital, Capital Medical University, NO.6 Tiantan Xili Dongcheng district, Beijing 100050, P R China; 2 Beijing Neurosurgical Institute, Capital Medical University, NO.6 Tiantan Xili Dongcheng district, Beijing 100050, P R China

**Keywords:** Germinoma, Medulla oblongata, Treatment

## Abstract

An intracranial germinoma is a tumor that is sensitive to radiotherapy. As medulla oblongata germinomas are extremely rare, determining an accurate preoperative diagnosis is challenging. Two cases of medulla oblongata lesions were surgically treated, and a postoperative diagnosis of germinoma was determined in both of the cases. The tumor in one patient completely resolved after a treatment course consisting of surgical intervention, radiotherapy and chemotherapy; the other patient, who did not receive any type of adjuvant treatment after surgery, suffered from tumor relapse and died from pneumonia 8 months following surgery. A preoperative diagnosis of medulla oblongata germinoma is difficult because of the lack of specific clinical signs and symptoms. If the correct diagnosis is reached, patients can have a favorable prognosis with proper evaluation and treatment. An invasive operation can potentially lesion and impair the function of the medulla oblongata, which is fatal to the patient.

## Background

Intracranial germinomas constitute 50 to 60% of central nervous system germ cell tumors and are commonly found in the suprasellar, basal ganglia, and pineal midline structures of the brain [1]. Medulla oblongata germinomas are particularly rare [2-5]. The medulla oblongata contains the cardiac, respiratory, vomiting and vasomotor centers of the human body; it is responsible for modulating the autonomic, involuntary functions, such as breathing, heart rate and blood pressure. There is considerable risk involved whenever surgery involves a medulla oblongata germinoma. An accurate preoperative diagnosis is crucial for prognosticating outcomes for patients because this tumor is sensitive to radiotherapy and chemotherapy [6,7]. In this study, we report two rare cases of primary medulla oblongata germinoma.

## Case presentation

### Case 1

**Figure 1:**
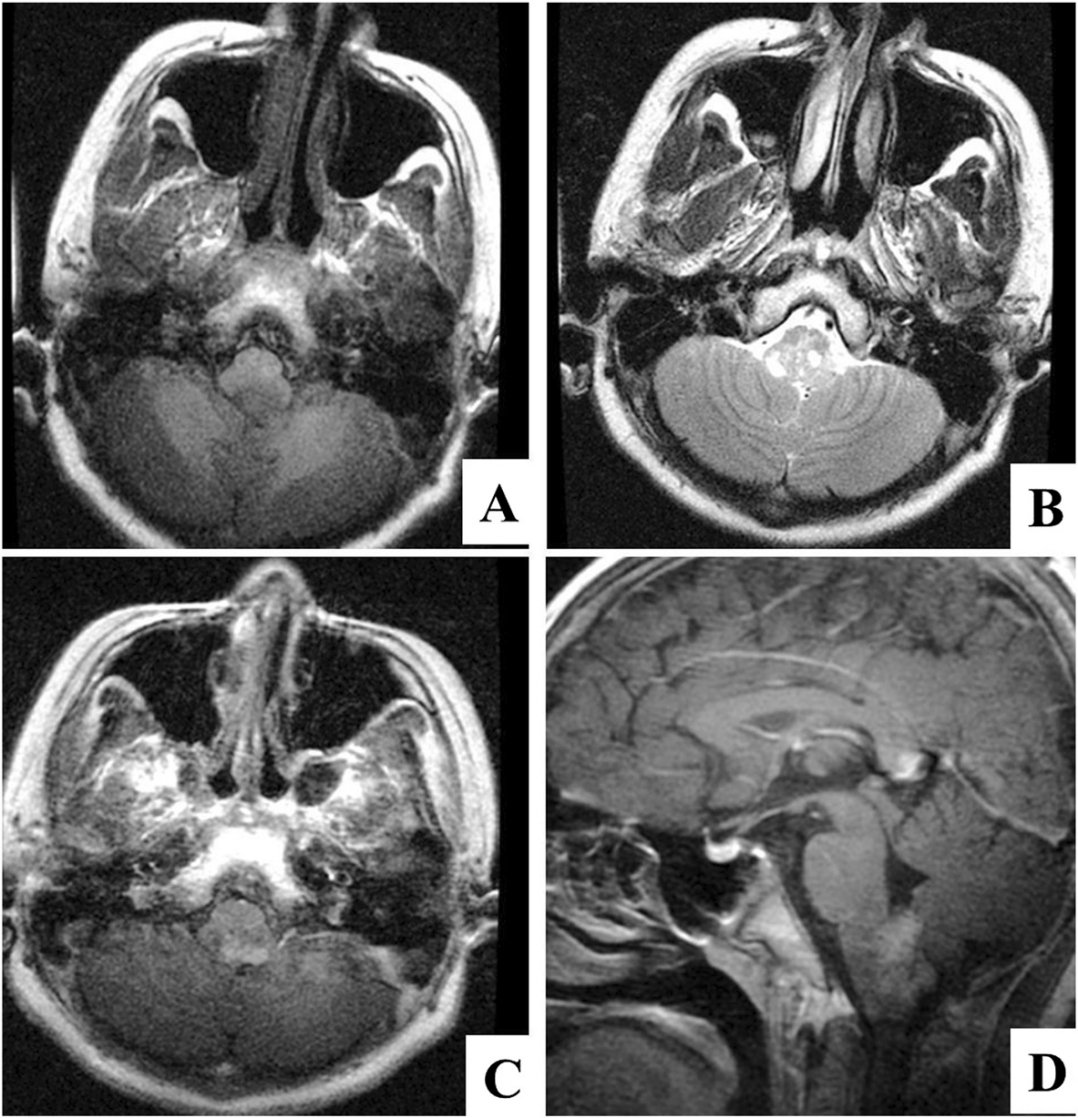
**Magnetic resonance imaging (MRI) scans of the case.** Axonal brain MRI shows a lesion in the dorsal medulla oblongata with low T1-weighted **(A)** and high T2-weighted signals **(B)**. Contrast-enhanced axial **(C)** and sagittal **(D)** T1-weighted images show the contrast-enhanced lesion.

A 14-year-old male patient presented with right facial numbness and gait instability lasting 6 months. The neurological examination upon admission revealed right-sided deficits in cranial nerves V, VIII and IX. The magnetic resonance imaging (MRI) showed a lesion in the dorsal region of the medulla oblongata (Figure 1). Our main diagnosis in the differential for this lesion was brain stem glioma. The cerebrospinal fluid (CSF) examination was not performed preoperatively.

A subtotal resection of the lesion was performed using a suboccipital midline approach. The tumor was pinkish, soft, and irregular; it originated from the obex of the medulla oblongata and extended into the fourth ventricle.

**Figure 2:**
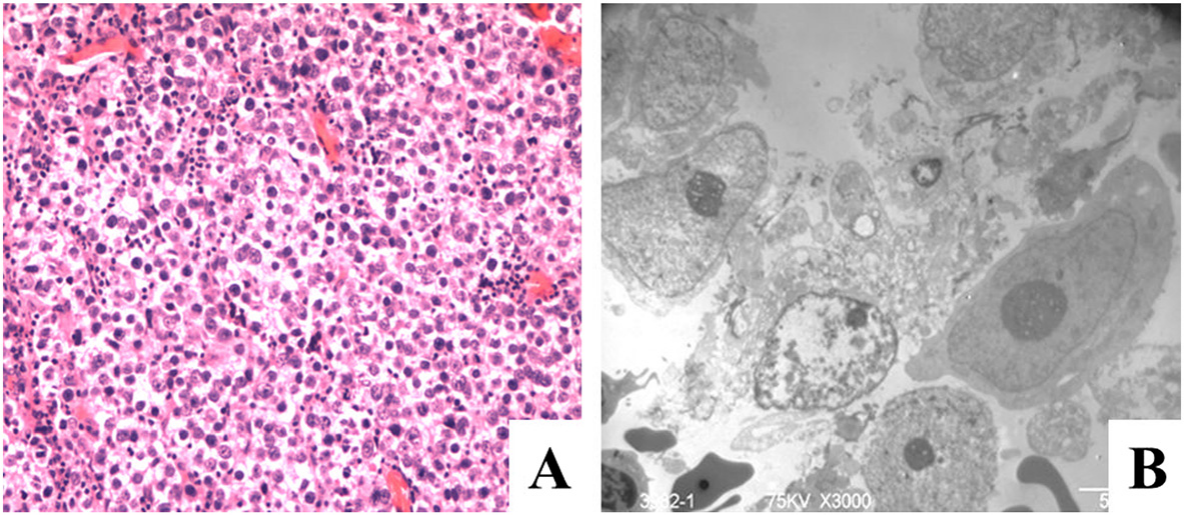
**Pathological results of the specimen.** Histopathological analyses reveal large spheroidal cells with oval nuclei (H&E, ×100) **(A)**. Under transmission electron microscope, the nucleolus of some tumor cells were prominent (arrowhead) and lymphocytes were found scattered between tumor cells (*) **(B)**.

**Figure 3:**
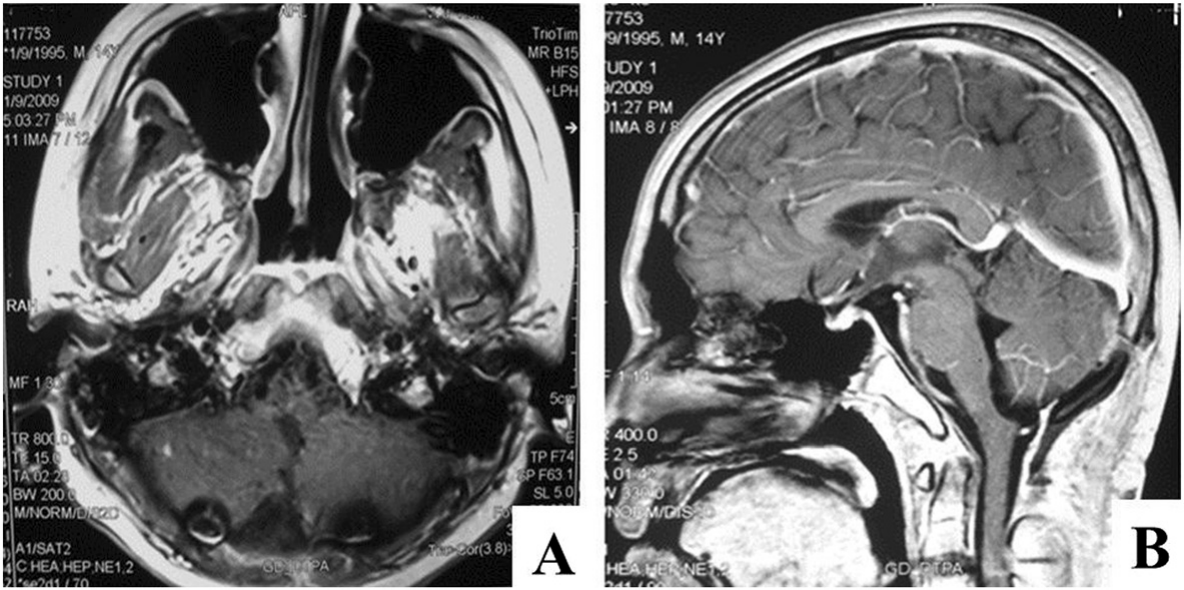
Axonal (A) and sagittal (B) brain magnetic resonance imaging (MRI) scans 6 months after surgery (2 months after chemotherapy and radiotherapy) demonstrating image-complete resection.

The postoperative course was uneventful except for transient dysphagia. The histopathological analysis confirmed the diagnosis of germinoma (Figure 2). The patient was treated by gamma knife and subsequently started on two rounds of PEB (cisplafin, etoposide and bleomycin) chemotherapy. The patient returned to school 3 months after surgery (Figure 3). He was followed up for 4 years and has not experienced any tumor recurrence.

### Case 2

**Figure 4:**
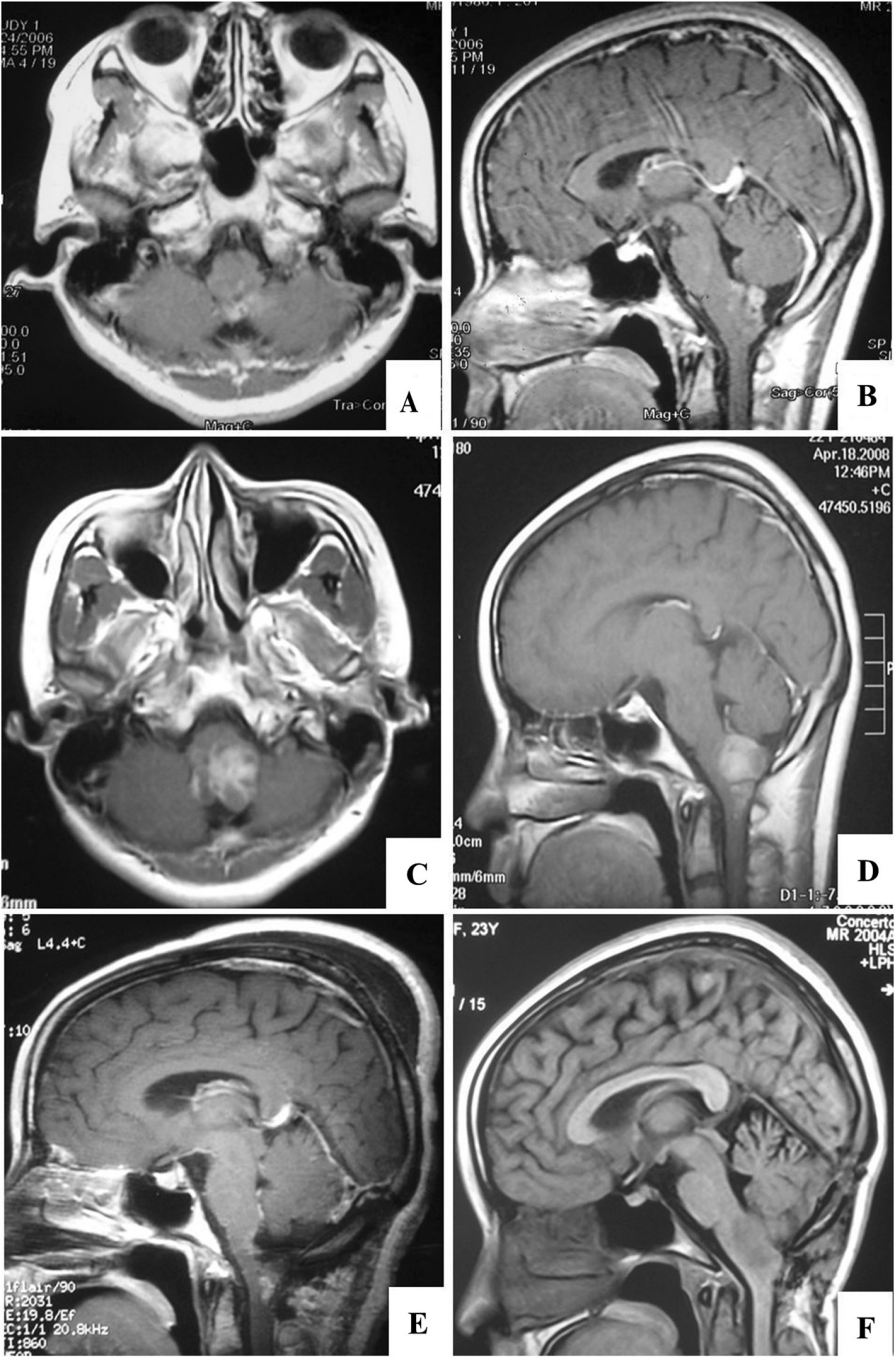
**Brain magnetic resonance (MRI) scans of the case.** Axonal **(A)** and sagittal **(B)** T1-weighted brain MRI 5 years before gamma knife treatment, depicting an enhancing lesion in the dorsal medulla oblongata. Preoperative MRI scans showing growth of the tumor **(C**, **D)**. Post-operative brain MRI (10 days after surgery) demonstrating complete resection of the lesion **(E)**. MRI scans 7 months after surgery, demonstrating recurrence of the tumor **(F)**.

A 22-year-old female patient complained of dizziness for 5 years, right-handed numbness for 2 months and dysphagia for 2 months. The patient was diagnosed as having a brain stem tumor 5 years prior and was treated by gamma knife (Figure 4A-B) at another hospital. The symptoms were effectively managed within a short time period. The neurological examination upon admission showed nystagmus and the absence of a gag reflex. The T1 and T2 MRI revealed a cystic lesion in the dorsal region of the medulla oblongata with areas of mixed signal intensities (Figure 4C-D). The serum and CSF alpha-fetoprotein (AFP) and beta-hCG (human chorionic gonadotropin) were normal. The preliminary diagnosis was brain stem glioma.

A subtotal resection of the lesion was performed using a suboccipital midline approach (Figure 4E). The tumor originated from the obex of the medulla oblongata and was a brown mass with a solid/cystic consistency that was prone to bleeding.

The postoperative course was complicated by several factors. First, the patient required nasogastric feeds due to difficulties with swallowing. Second, the patient could not lie down but sustained labored breathing. Given the patient’s history of prior radiotherapy, we abstained from treating this patient with another course of radiotherapy for fear of provoking brain stem edema. Due to the patient’s ominous clinical condition, his family elected to postpone chemotherapy treatment. The tumor relapsed 7 months following surgery and was confirmed by MRI (Figure 4F). The patient died from pneumonia in the eighth month following surgery.

## Discussion

Intracranial germinomas are rare tumors that are most commonly found in children and that account for 0.5 to 2% of primary intracranial neoplasms [1,4]. There is a higher incidence of intracranial germinomas in Eastern Asian populations than in those from Western countries. Germinomas are usually found in midline brain structures, most commonly in the pineal and suprasellar regions, and less frequently in the basal ganglia and thalamus [1-5,7-12]. The pathogenesis of intracranial germinomas was thought to be due to the entrapment of migrating totipotential cells during the early period of rostral neural tube development [7]. Nakajima suggested that the female predominance of tumors in the neurohypophysis and medulla and the male predominance of germinomas in the pineal region may be attributed to a delayed closure of the anterior neuropore in females compared with males [11]. The clinical manifestations of germinomas largely depend on the intracranial location of the tumors. Approximately 60% of germinomas originate in the pineal region, an area that shows male predominance. Patients with germinomas that grow in this specific location classically present with a headache, hydrocephalus and Parinaud’s syndrome [10]. Approximately 30% of the tumors originate from the suprasellar region, an area that shows a slight female predominance. The main symptoms of germinomas in this location are polydipsia, polyuria, visual impairment and other hypothalamic-pituitary dysfunctions, which include growth retardation and delayed puberty. Germinomas originating from the basal ganglia and thalamus account for approximately 4 to 10% of intracranial germ cell tumors [10]. With regard to these types of tumor locations, most patients tend to be male and suffer from early symptoms of paralysis, dysnoesia and dyskinesia. Intracranial germinomas have predilections for certain sites and demonstrate a pattern of gender preference. This cancer does not yet have specific tumor markers from which to prognosticate outcomes, which is different from other types of intracranial germ cell tumors [2,5,8,9].

Primary medullary germinomas are extremely rare, which makes preoperative diagnosis difficult. According to the literature retrieval, only 15 reported cases were included this study (Table 1) [2-5,8,9,11-15] and all of the reported cases were from Eastern Asia. The primary medullary germinoma has an obvious gender bias (male:female=2:3). The average age of onset of intracranial germinoma is 16.1 years, whereas the average onset of primary medullary germinoma occurs later, at an average age of 24.2 years (range 12 to 40 years). There was only one case in which the patient had suffered from Klinefelter syndrome [14]. The delayed closure of the anterior neuropore during female embryonic development may account for the female predominance in primary medullary germinomas.

**Table 1:** Summary of reported cases of primary medulla oblongata germinoma

**Number**	**Author (year) [reference]**	**Age, years**	**Sex**	**Symptoms**	**Treatment**	**Outcome**	**Follow-up period**
1	Poungvarin [15]	17	M	Pneumonia and presented with intermittent apnea	S, RT	Died of pneumonia	92 days
2	Hashimoto [14]	19	M	Klinefelter syndrome, CN IX, X paresis, sleep apnea	Biopsy, WB+WS RT	CR	2 months
3	Sugiyama [12]	32	F	CN VII, IX, XI, XII paresis, ataxia	PR, Local+WS RT	CR	9 years
4	Israel (1996) [8]	40	F	Headache, vomiting, cerebellar signs	STR, CMT, FB RT	CR	18 months
5	Nakajima [11]	18	F	Hiccups, nystagmus	PR, CMT, GKS	CR	8 months
6	Yoshida (2003) [8]	33	F	CN V, VI, VII paresis	STR, CMT	CR	7 months
7	Yen [5]	16	F	Static and kinetic ataxia	STR, FB+WB+WS RT	CR	7 years
8	Yang (2009)[8]	12	M	CN IX, X, XII palsy, lethargy, loss of appetite	PR, CMT, RT	CR	6 months
9	Akimoto [4]	30	F	Left CN VI palsy, bilateral palsy of CNs IX, X,XII	TR, CMT, FB RT	CR	1 year
10	Akimoto [4]	24	M	Headache	PR, CMT, FB RT	CR	8 months
11	Yasuhara [3]	27	F	CN VIII, IX, X,XII paresis, ataxia, sleep apnea, numbness of limbs	Biopsy, CMT, RT	CR	6 months
12	Shuto [2]	28	M	Loss of balance	STR, CMT, FB+WS RT	CR	3 years
13	Nakatsuka [8]	31	F	Hiccups, hoarseness, and swallowing disturbances	STR, CMT, FB RT	CR	6 months
14	This study	14	M	CN VII VIII, IX paresis, nystagmus	STR, GKS, CMT	CR	4.5 years
15	This study	22	F	CN IX XI paresis, nystagmus, numbness of limbs	GKS, STR	Died of pneumonia	8 months

M, male; F, female; CMT, chemotherapy; CN, cranial nerve; CR, complete remission; FB, focal brain; GKS, gamma knife surgery; S, surgery; STR, subtotal resection; PR, partial resection; TR, total resection; WB, whole-brain irradiation; WS, whole-spine irradiation.

Compared with other medullary lesions, there are no significant differences in the clinical presentation of primary medullary lesions. Dysphagia, cough, and limb sensory-motor disorders are the most common symptoms. Patients may often complain of headache, impaired vision, and hiccups among other symptoms. Reviewing the brain CT and MRI scans is very important for diagnosing brain stem germinomas. Intracranial germinomas show hyperdense signaling on brain computer tomography (CT) [1,4,11]. With regard to MRI evaluation, brain stem germinomas have a relatively clear boundary, are iso-intense in T1-weighted (T1WI) imaging and show long T2 signal intensities. In contrast-enhanced brain CT and MRI, the tumor usually shows homogeneous enhancement. The primary medullary germinoma often has a base from the bottom of the fourth ventricle to approximately the midline of the first cervical spinal cord and protrudes into the fourth ventricle. The differential diagnoses for primary medullary germinomas are ependymomas and gliomas. Ependymomas, which mostly occur in children, originate from ependymal cells lining the fourth ventricle and generally grow from within the brain stem. Ependymomas that stem from the fourth ventricle often lead to obstructive hydrocephalus. In 50 to 80% of patients, calcification can be observed on CT. In general, brain MRI shows long T1 and T2 signals with obvious enhancement. If there are cystic changes or calcifications within the ependymoma, homogenous signals would show on brain MRI. Medullary exophytic gliomas are low-grade astrocytomas in the majority of cases, which are hyperintense on TIWI and have heterogeneous signal intensity with T2WI. Additionally, patients with this pathology would demonstrate thickening of the brain stem and cystic changes on neuroimaging of the medulla area. Tumor markers, such as positive AFP or beta-hCG in the blood and cerebrospinal fluid concentrations of patients would also be helpful in clinching the preoperative diagnosis of medullary germinoma.

There is currently no standard treatment regimen for intracranial germinoma. We know that germinomas are sensitive to radiotherapy. One series reported that patients who were treated with radiotherapy for intracranial germinoma had 10-year survival rates of 90% [6,7]. Considering that germinomas mostly occur in pediatric patients, there is the potential for many long-term side effects after radiotherapy at such early ages, such as skeletal development retardation, cognitive dysfunction, and hypopituitarism. Therefore, many scholars choose to administer small doses of radiation with chemotherapy, a successful combination that has also proven to yield favorable long-term effects [1,6,7].

Experimental radiotherapy is the preferred treatment option for intracranial germinoma originating in the pineal, suprasellar or basal ganglia regions. However, because primary medullary germinoma is extremely rare, it is challenging to correctly diagnose this disease preoperatively based on the clinical presentation and imaging reports. In all of the reported cases of patients with primary medullary germinoma who underwent surgery, 86.7% (13/15) had a favorable prognosis [2-5,9,11-14]. Furthermore, Sugiyama and Yen performed partial resections on primary medullary germinomas, followed by adjuvant local radiotherapy of the posterior fossa and whole spinal cord radiotherapy [5,12]. The tumors completely resolved following this treatment. They reported no tumor recurrences after a 9- and 7-year follow-up period. Other patients in the medical literature with primary medullary germinomas who underwent combination radiotherapy and chemotherapy also achieved good therapeutic effects. The medulla oblongata has nuclei, which regulate vomiting, swallowing, coughing and sneezing. Following the operation for germinoma, the patients almost always complained of dyspnea and dysphagia. Similar to the reviewed cases in this study, two patients also died of pneumonia. These deaths were related to the dysfunction of the medulla oblongata. Craniospinal irradiation (CSI) with or without chemotherapy is an effective salvage treatment for recurrence. Hu reported 5-year survival rates of 71 and 92.9% for all of the patients and for those receiving salvage CSI, respectively [6]. One of our patients, who had an early recurrence of the tumor, did not undergo timely adjuvant treatment and died from pneumonia.

## Conclusion

Primary medullary germinoma is extremely rare. A preoperative diagnosis is difficult due to the absence of specific clinical features. With proper evaluation and treatment of this disease, patients can have favorable prognoses.

## Consent

Written informed consent was obtained from the patient reported in case 1, and from the father of the patient in case 2 for the publication of this report.

## Abbreviations

AFP: Alpha-fetoprotein; hCG: Human chorionic gonadotropin; CSF: Cerebrospinal fluid; CSI: Craniospinal irradiation; CT: Computer tomography; MRI: Magnetic resonance imaging; PEB: Cisplafin, etoposide and bleomycin; T1WI: T1-weighted imaging.

## Competing interests

The authors declare that they have no competing interests.

## Authors’ contributions

SYH made substantial contributions to the study conception and design and the acquisition of the data. DL and SYH also drafted the manuscript and revised its final form. JF, LW, ZW and JTZ were involved in the analysis and interpretation of the data. LWZ contributed to the interpretation of the data and gave the final approval for the version to be published. All of the authors read and approved the final manuscript.
